# Engineered soluble ACE2 receptor: Responding to change with change

**DOI:** 10.3389/fimmu.2022.1084331

**Published:** 2023-01-18

**Authors:** Guangyao Li, Kewen Qian, Shuyi Zhang, Wenyan Fu, Jian Zhao, Changhai Lei, Shi Hu

**Affiliations:** ^1^ Department of Biophysics, College of Basic Medical Sciences, Naval Medical University (Second Military Medical University), Shanghai, China; ^2^ Department of Assisted Reproduction, Shanghai Ninth People’s Hospital, Shanghai Jiao Tong University School of Medicine, Shanghai, China; ^3^ KOCHKOR Biotech, Inc., Shanghai, China

**Keywords:** ACE2 decoy receptor, COVID-19, protein engineering, SARS coronavirus 2, therapeutic proteins

## Abstract

SARS coronavirus 2 (SARS-CoV-2) invades the human body by binding to major receptors such as ACE2 *via* its S-spike protein, so the interaction of receptor-binding sites has been a hot topic in the development of coronavirus drugs. At present, the clinical progress in monoclonal antibody therapy that occurred early in the pandemic is gradually showing signs of slowing. While recombinant soluble ACE2, as an alternative therapy, has been modified by many engineering methods, both the safety and functional aspects are approaching maturity, and this therapy shows great potential for broadly neutralizing coronaviruses, but its progress in clinical development remains stalled. Therefore, there are still several key problems to be considered and solved for recombinant soluble ACE2 to be approved as a clinical treatment as soon as possible.

## Introduction

Since the global outbreak of SARS-COV-2 in 2019, the world has faced unprecedented public health security challenges. Vulnerable individuals, including elderly individuals and those with underlying diseases, have been fatally hit by the virus pandemic. Hence, teams around the world have conducted intensive research and developed numerous therapeutic options for COVID-19, which mainly involves fever and pulmonary symptoms (1) and, less commonly, neurological disorders (2). It is now well established that the process of host invasion by SARS-CoV-2 is mediated by the interaction of the viral spike (S) protein, a trimeric protein, with angiotensin-converting enzyme 2 (ACE2) on the host cell membrane (3), implying that ACE2 is the main receptor for cell invasion by SARS-CoV-2. Therefore, the monoclonal antibody (mAb) drugs developed in the early stage of the pandemic, which focused on the contact point between the receptor-binding domain (RBD) of the S protein and ACE2 as the main drug target, not only effectively inhibited the expanding epidemic of the strain and alleviated the clinical symptoms but also played a certain degree of preventive effect (4). However, the evolution of endless variants, such as omicron, has led to immune escape and antigenic shift from the vast majority of monoclonal antibody therapies and the emergence of drug resistance (5). Even though the above situation was somewhat ameliorated by the subsequent proposed monoclonal antibody cocktail combination regimen (6), the ability of mAb therapy to address the dispersal of different antigenic coronaviruses of various wildlife origins is quite limited and unlikely to be broadly potent against all coronaviruses. Therefore, soluble ACE2 was created as an alternative therapy.

ACE2 is homologous to ACE, which mainly acts on angiotensin II and plays a key role in fluid homeostasis. In the renin-angiotensin system, ACE2 hydrolyzes angiotensin II to Ang 1-7 to mitigate its proinflammatory effects, including changes in vascular smooth muscle and its permeability (7). In fact, the development of soluble ACE2 (sACE2) began with the study of the pathogenesis of ARDS, as the Ang II-based anti-inflammatory effects were effective in improving lung injury and hypoxemia and resolving homeostatic imbalances in related signaling pathways in in a piglet model of ARDS (8). Today, recombinant soluble ACE2 is being reconsidered for its potential use as an alternative treatment strategy for SARS-CoV-2-infected patients, and several research groups have verified the blocking effect of recombinant soluble ACE2 on the early infection of SARS-CoV-2 in preclinical tests such as human engineered organs, which is expected to promote the next clinical progress of rsACE2 (9)., focusing on the degree of improvement in pulmonary and renal symptoms and the efficacy and safety of the drug.

The main advantage of recombinant soluble ACE2 is its breadth. In principle, even if a mutant coronavirus becomes resistant to the neutralization of sACE2 due to antigenic drift, its affinity for the host’s natural ACE2 can hardly be maintained at the original level, leading to a decrease in the virulence of the strain (10). Thus, although the decoy receptor is inevitably mutated at the point of interaction compared to the natural ACE2 after being optimized by various engineering means, giving the viral spike variants the opportunity to effectively recognize and distinguish between the two to generate resistance, based on the aforementioned principle, if the virus readily mutates against the engineered ACE2 to escape neutralization, the price it pays may be a decrease in its affinity for the host cell’s natural receptor, implying the loss of most of its infectivity and virulence. Second, the ACE2 decoy receptor theoretically retains neutralizing potency against other coronavirus-associated strains and variants, suggesting its potential to address viral spillover in animal reservoirs, such as bats and pangolins, and thus as a powerful tool for future zoonotic coronavirus pandemics. The final possible advantage is that while mAb therapies are more mature in reducing viral load than sACE2, the catalytically active sACE2 is more effective in neutralizing infection and directly alleviating clinical symptoms in patients with COVID-19 (11), based on a series of the anti-inflammatory physiological effects of sACE2 on angiotensin II in the renin-angiotensin system, and protects patients’ lungs and heart. However, this dual action hypothesis has not yet been tested experimentally.

**Figure 1:**
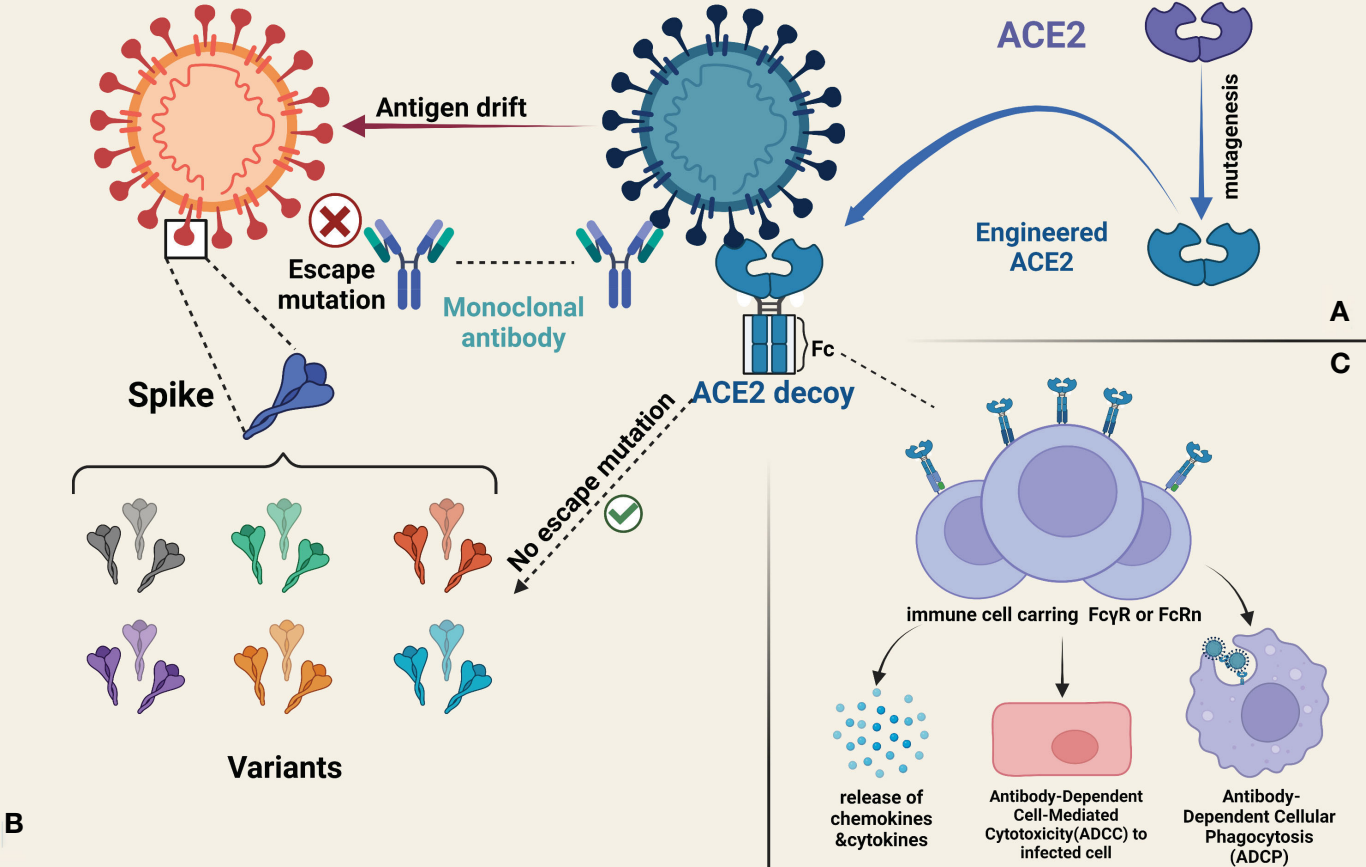
**(A)** rsACE2 exhibits superior affinity by means of mutagenesis and achieves stronger neutralization ability against virus mutant strains. **(B)** The development of monoclonal antibodies is limited to a single viral strain, so current monoclonal antibodies may lose the majority of their neutralizing ability in the face of diverse mutant strains. In contrast, the research of rsACE2 decoy recepor has shown that it can neutralize almost all of the SRAS-CoV-2-associated variants, preventing immune escape from occurring. **(C)** The fusion with the Fc structural domain of the antibody not only brings superior affinity and better pharmacokinetic properties to rsACE2 decoy recepor, but also the so-called Fc effector confers important functions such as modulating the degranulation or release of small particle chemicals like cytokines, NK cell-mediated ADCC function, and macrophage-mediated ADCP in immunomodulation.

To date, several teams have engineered soluble ACE2 decoy receptors by mutagenesis and Fc fusion to cover a full range of viral evolution in terms of affinity, pharmacokinetics, effector function mutation and multivalent binding in an attempt to meet the evolving needs of clinical care (**Figure 1**).

As an optimized therapeutic alternative to monoclonal antibodies, a rather critical aspect of sACE2 optimization is to maintain consistent or even tighter affinity with the former in terms of competitive neutralization of the virus. To achieve the affinity level of less than 10 nanomoles between sACE2 and SARS-CoV-2 that has been stably achieved by established monoclonal antibodies (12), point mutagenesis and broad screening of WT sACE2 with poor affinity levels were performed with various engineering techniques. There are teams who have explored the potential sequence mutation possibilities of ACE2 by deep mutation scanning techniques; by building a large-scale dataset of all individual amino acid substitutions in ACE2, covering the active regions recognized by ACE2 and the RBD; and finally by binning and screening out the tightly bound mutant units among them, which can achieve monomeric affinity enhancement to the RBD of SARS-CoV-2 at the picomolar level (13, 14). Zahradník J et al. also employed directed evolution, a classic and practical protein engineering tool, to mimic the evolutionary pathway of the natural strain SARS-CoV-2 and artificially accelerated the evolutionary process considerably, illustrating the importance of viral RBD-host ACE2 affinity for virulent strain variants to evolve a higher infectious advantage, laterally confirming the necessity of rsACE2 affinity optimization (15).

The aforementioned recombinant soluble ACE2 developed for ARDS patients, although promising in alleviating clinical symptoms such as hypoxemia and protecting the lungs, has rather limited efficacy, probably due to its short half-life of ~3 hours in humans (16), resulting in an inability to accumulate sufficient concentrations of the drug at the lesion site. The half-life stability of recombinant protein drugs can be satisfactorily enhanced by the fusion of the extracellular structural domain with the Fc segment of human immunoglobulin G (IgG) (17). For instance, TIGIT-Ig (18), a recombinant fusion protein of the T-cell immune receptor with the Ig and ITIM structural domains that showed excellent stability both *in vivo* and ex vivo while maintaining its function, and ACE2-Ig, a recombinant fusion protein fused to the Fc region of IgG, which also achieved the expected half-life improvement consistent with the former in pharmacokinetic-related experiments *in vivo*. Interestingly, further *in vitro* neutralization experiments showed that ACE2-Ig maintained full peptidase activity and had higher affinity for SARS-CoV-2 spike-in protein (19). Not coincidentally, other teams have also actively advanced experiments on the treatment of Ig fusion proteins in COVID-19 animal models (20, 21).

In addition, the Fc region of the antibody has the ability to trigger both viral clearance and killing of infected cells as well as immune functions such as cytokine release and antigen presentation, an effector process induced by binding to FcRn or FcγR, called the Fc effector, which has been demonstrated in the development of monoclonal antibodies against SARS-CoV-2 (22). However, for some antibodies, the recognition of bound viral epitopes carries an increased risk of infection and virulence, called antibody-dependent enhancement (ADE) (23). However, no *in vivo* experiments on infected hosts have yet found that recombinant sACE2 enhances the likelihood of cellular infection. In other words, the proven promising clinical therapeutic efficacy of soluble decoy receptor drugs makes ADE an insufficient barrier to limit its development. What is currently outstanding in this field is the applicability of various novel enhanced fusion Fc effectors for the treatment and prophylaxis of newly diagnosed patients. On the other hand, another promising direction to highlight in Fc fusion is the integration of the concept of polyvalent therapeutics into the integrated design of soluble decoy receptors. The importance of polyvalent drugs in the response to viral safety and health problems has always been unquestionable, as it is widely recognized that polyvalent drugs have more efficient preventive and therapeutic efficacy than monovalent drugs (24, 25). For example, trivalent sACE2 (26), which binds closely to the viral spike protein trimer, and higher-valent sACE2-Fc, such as ACE2-Fc-TD (27), a tetrameric ACE2 protein that shows a more advantageous IC_50_ for viral neutralization than dimeric ACE2-Fc, both have excellent potential for long-term development after addressing issues such as design balance and production feasibility.

Reports on the development of a recombinant soluble ACE2 drug for COVID-19 were published as early as two years ago. Unfortunately, to date, only a few clinical trials have been approved. According to the data published by NIH, NCT04335136 and NCT05065645 were completed to validate the effects of two different forms of rsACE2 drugs for patients with COVID-19, intravenous injection and nebulized inhalation, respectively, and only the former submitted the trial results, which were not specific. Although the development of recombinant soluble ACE2 is progressing at a brisk pace, it has not yet been able to advance in clinical trials, which would require addressing several key issues identified in the current study. (1) The progress of affinity engineering mutagenesis of sACE2 is promising, but an important issue that cannot be ignored is the equilibrium of the process of achieving high affinity. For example, there is a high risk of artificial bias and overneutralization in the design of high-affinity decoy receptors for targets *in vitro*, thus neglecting other properties such as stability, safety, and generalizability that need to be balanced for the sake of achieving tight neutralization, which can be unaffordable. (2) Furthermore, as clinical treatment dosages are greatly increased compared to experimental measures, the question of whether the intrinsic enzymatic activity of sACE2, which can directly relieve patients’ symptoms better than monoclonal antibodies as previously mentioned, may become a safety risk at high dosages must be taken into account, since excessive conversion of Ang II may cause a series of hemodynamic problems. Therefore, there is an urgent need to find the optimal option for a powerful, stable and safe method for universal elimination of intrinsic enzyme activity. (3) The complex and numerous Fc fusion effectors enable and improve various functions of recombinant receptors, and at the same time, the problems of modulation and orientation come with them. The optimal Fc fusion strategies suitable for COVID-19 treatment, including effect enhancement or multifunctional effectors, such as FcRn or FcγR, have yet to be selected and confirmed. For example, Lieberman et al. reported a possible mediating role of the FcγR receptor in the mechanism of host systemic inflammation triggered by SARS-CoV-2 (28). These similar studies emphasized the need for safety as the first point of consideration for the development of rsACE2 drugs, an important aspect of which is an in-depth understanding of the Fc fusion structural domain and its optimal modification. Fortunately, both humanization bias and safety-based immunogenicity issues can be predicted and guided by computational models to rule out high-risk choices or eliminate enzyme activity, and artificial domain technologies with high learning capabilities and deep abstraction design are playing an increasingly important role (29–31).

At a time when progress in monoclonal antibody and vaccine therapies is stalled in the face of coronavirus variants that continue to mutate and evolve, engineered soluble ACE2 offers new ideas to overcome the challenge of antigenic drift, paves a broad path for future pandemics due to zoonotic coronavirus spillover, and is undoubtedly a promising strategy in the face of current and future public health problems. Fortunately, there are still numerous opportunities to continually refine and evolve this device as we accumulate ongoing clinical experience.

## Author contributions

SH, CL, and GL contributed to conception and design of the study. WF and JZ organized the database. KQ and JZ performed the statistical analysis. GL wrote the first draft of the manuscript. All authors contributed to manuscript revision, read, and approved the submitted version.
